# Therapy of prostate cancer using a novel cancer terminator virus and a small molecule BH-3 mimetic

**DOI:** 10.18632/oncotarget.3544

**Published:** 2015-03-12

**Authors:** Siddik Sarkar, Bridget A. Quinn, Xue-Ning Shen, Rupesh Dash, Swadesh K. Das, Luni Emdad, Alexander L. Klibanov, Xiang-Yang Wang, Maurizio Pellecchia, Devanand Sarkar, Paul B. Fisher

**Affiliations:** ^1^ Department of Human and Molecular Genetics, Virginia Commonwealth University, School of Medicine, Richmond, VA, USA; ^2^ Institute of Life Sciences, Chandrasekharpur, Bhubaneswar, Orissa, India; ^3^ VCU Institute of Molecular Medicine, Virginia Commonwealth University, School of Medicine, Richmond, VA, USA; ^4^ VCU Massey Cancer Center, Virginia Commonwealth University, School of Medicine, Richmond, VA, USA; ^5^ Division of Cardiovascular Medicine and Department of Biomedical Engineering, University of Virginia, Charlottesville, VA, USA; ^6^ Infectious and Inflammatory Disease Center, Cancer Center, Sanford-Burnham Medical Research Institute, La Jolla, CA, USA

**Keywords:** BH3 mimetic, cancer terminator virus (CTV, prostate cancer (CaP), truncated CCN1 (tCCN1)-Prom, PEG-Prom

## Abstract

Despite recent advances, treatment options for advanced prostate cancer (CaP) remain limited. We are pioneering approaches to treat advanced CaP that employ conditionally replication-competent oncolytic adenoviruses that simultaneously produce a systemically active cancer-specific therapeutic cytokine, *mda-*7/IL-24, *Cancer Terminator Viruses* (*CTV*). A truncated version of the *CCN1/CYR61* gene promoter, tCCN1-Prom, was more active than progression elevated gene-3 promoter (PEG-Prom) in regulating transformation-selective transgene expression in CaP and oncogene-transformed rat embryo cells. Accordingly, we developed a new *CTV*, Ad.tCCN1-*CTV-m*7, which displayed dose-dependent killing of CaP without harming normal prostate epithelial cells *in vitro* with significant anti-cancer activity *in vivo* in both nude mouse CaP xenograft and transgenic Hi-*Myc* mice (using ultrasound-targeted microbubble (MB)-destruction, UTMD, with decorated MBs). Resistance to *mda-*7/IL-24*-*induced cell deathcorrelated with overexpression of Bcl-2 family proteins. Inhibiting Mcl-1 using an enhanced BH3 mimetic, BI-97D6, sensitized CaP cell lines to *mda-*7/IL-24*-*induced apoptosis. Combining BI-97D6 with Ads expressing *mda-*7/IL-24promoted ER stress, decreased anti-apoptotic Mcl-1 expression and enhanced *mda*-7/IL-24expression through mRNA stabilization selectively in CaP cells. In Hi-*myc* mice, the combination induced enhanced apoptosis and tumor growth suppression. These studies highlight therapeutic efficacy of combining a BH3 mimetic with a novel *CTV*, supporting potential clinical applications for treating advanced CaP.

## INTRODUCTION

Prostate cancer (CaP) is the leading cause of cancer deaths in men in the USA despite improvements in chemo-, radio-, and hormonal-therapies. The 5-year survival of CaP patients with loco-regional disease is > 95%, but survival rates decrease dramatically to < 28% when CaP has metastasized to bones and distant organs [1]. For that reason, it is imperative to find a means of preventing or treating CaP after it has metastasized. Some success in treating advanced CaP patients with bone metastases has been achieved using external beam radiation therapy, hormonal-therapy and chemotherapy with bisphosphonates, which target bone remodeling [2]. Although the effectiveness of these combination treatment strategies appeared promising they were limited to the areas of bone they target and were ineffective at later stages of metastasis. Additionally, the side effects of current treatment approaches mandate innovative and improved strategies to treat CaP, especially advanced CaP.

Viral-based gene therapy is currently considered an attractive tactic to treat various cancers. However, since CaP is a relatively slow growing disease, it may require repeated gene therapy applications over the life span of the patient to be effective. In these contexts, conditionally replication competent adenoviruses (Ads) (CRCA) that selectively induce cytolysis in prostate cancer cells with concomitant production of a therapeutic gene represent a potential treatment option for patients with CaP, including those with metastatic disease. The therapeutic potential of oncolytic Ads or CRCAs has been evaluated in human clinical trials [3, 4]. They were found to be safe and well tolerated in patients with advanced cancers [5, 6], although therapeutic efficacy as single agents was not promising. The route of administration of oncolytic viruses has predominantly involved intratumoral injection [7], and restricted effectiveness potentially resulted from limited intratumoral spread from the injection site [8].

Therapeutically armed bipartite conditionally-oncolytic Ads, such as Ad.PEG-*E1A-mda-*7 (Ad.PEG-*CTV*-*m*7 or Ad.*CTV-m*7) [9-12] in which replication is controlled by the progression elevated gene-3 promoter (PEG-Prom) resulting in simultaneous production of *mda-*7/IL-24, have wider applications for cancer therapy showing enhanced activity in killing cancer cells *in vivo* as compared to single conditionally-oncolytic Ads [9, 13, 14]. The improved therapeutic potential of Ad.*CTV-m*7 is mediated by the *mda*-7/IL-24 transgene, which when translated produces and secretes MDA-7/IL-24 protein that induces “bystander” [15, 16] cancer-specific cytotoxic effects eliminating both primary transduced tumor cells as well as adjacent non-transduced tumor cells and distant metastases [11, 12, 15, 17]. *In vitro* cell culture and *in vivo* pre-clinical animal studies support the use of *CTV-m*7 as a potential reagent to treat local as well as advanced CaP [11-13], providing proof-of-concept for *CTV* as an efficacious reagent for cancer therapy.

The *CCN1/CYR61* gene displays elevated expression as a consequence of oncogenic transformation in various cancers including CaP [18, 19], and expression increases with aggressiveness of the transformed cells [19, 20]. Regulation is predominantly controlled at a transcriptional level [19, 21]. Based on these considerations, we evaluated the CCN1-Prom for cancer-selective expression and identified a truncated version of this promoter (tCCN1*-*Prom) as a potential genomic reagent to develop conditionally replication-competent Ads. Ads were engineered in which the tCCN1-Prom controls Ad E1A expression and the constitutive cytomegalovirus promoter (CMV*-*Prom) controls *mda-*7/IL-24 expression resulting in a new class of *CTV*, i.e., Ad.tCCN1*-CTV*-*m*7).

To optimize the utility of viruses for gene therapy it is essential to develop approaches to systemically administer these agents in a manner that limits non-specific trapping in non-target organs (such as the liver) and elimination by the immune system. To address these impediments to effective gene therapy we have developed a “stealth delivery approach”, called ultrasound-targeted microbubble-destruction (UTMD), to systemically deliver viruses such as *CTV-m*7 [12, 22, 23]. Further improvements in tumor-specific delivery can be achieved using MBs by functionalizing with linkers or ligands to form targeted or decorated MBs (D-MBs) [24, 25] to specifically bind to the tumor vasculature and inflamed tissues around the tumor. In principle, D-MBs coupled with the UTMD approach will provide an effective means of releasing the *CTV-m*7 payload specifically at the tumor region following sonoporation [22-25] leading to oncolysis at the site of direct infection and *mda*-7/IL-24-induced cell death in adjacent and distant tumor cells.

Considering the slow growing and progressive nature of CaP and also the development of resistance to conventional treatment with a single agent, employing a combinatorial approach with agents affecting different cancer-specific pathways may be required to effectively treat advanced CaP. Moreover, it was found that ectopic expression of Mcl-1 or Bcl-2 family of proteins in prostate cancer cells led to resistance to *mda*-7/IL-24-induced cell death [23]. The Bcl-2 family of proteins is commonly over-expressed in prostate cancers especially in advanced hormone refractory prostate cancer [26]. BH3 mimetics, which function as inhibitors of the anti-apoptotic Bcl-2 proteins, were able to sensitize CaP cells to chemotherapy [23, 27]. To further validate this hypothesis, it is necessary to confirm cancer-selective activity in appropriate pre-clinical animal models, including those with a compromised immune system (permitting growth of human CaP cells) and immune-competent genetically engineered transgenic mouse models of CaP. We now demonstrate that CTV-*m*7 (Ad.tCCN1-*CTV-m*7) delivered by a UTMD approach using D-MBs in combination with small molecule inhibitors of Mcl-1 (e.g., BI-97C1 and BI-97D6) [27, 28], promotes CaP regression in multiple pre-clinical animal models. These provocative observations provide a path for potentially translating this combinatorial approach and innovative therapeutic strategy into the clinic for the treatment of advanced hormone refractory CaP.

## RESULTS

### Construction and characterization of Ad.tCCN1-*CTV-m7*

**Figure 1:**
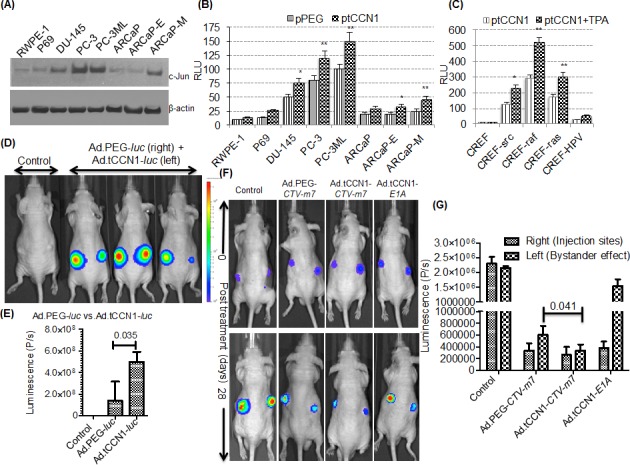
Utilizing the cancer-selective tCCN1-Prom to make conditionally replication-competent bipartite Cancer Terminator Virus (Ad.tCCN1-*CTV-m*7) A. Western blotting of whole cell lysates of CaP cells. B. CaP cells were transfected with pGL3-*luc* reporter vector driven by *PEG-*Prom (pPEG-*luc*) or *tCCN1*-Prom (ptCCN1-*luc)*. pRL-TK (Renilla luciferase) was co-transfected for the normalization of luciferase activity, and the luminescence readings were plotted as relative luminescence units (RLU). * (p<0.05) and ** (p<0.01) indicate statistical significance as determined by using t-test between pPEG-*luc* and ptCCN1-*luc* transfected cells. C. CREF and transformed CREF cells containing a single oncogene were transfected with ptCCN1-*luc* and/or TPA and RLU were measured. D, E. Ad.PEG-*luc* and Ad.tCCN1-*luc* were injected i.t. into the right and left flank, respectively, in DU-145 bearing tumor xenografts in male athymic nude mice followed by monitoring using BLI. Quantification of image signals were generated using an IVIS spectrum coupled with Living Image 4.3.1. F. Ad.PEG-*CTV-m*7, Ad.tCCN1-*CTV-m*7 or Ad.tCCN1-*E1A* was injected i.t. into the right flank of DU-145-*luc* bearing tumor xenografts in male athymic nude mice as described in Materials and Methods. BLI imaging at time 0 (prior to first treatment) and after 28-days (7 day after last treatment). G. Image analyses showing antitumor response in the right flank bearing tumor (primary Ad.*CTV-m*7 injected site) and left flank (secondary site not directly infected but showing a “*bystander effect*”).

*CCN1/CYR61* is an early response gene regulated transcriptionally in a protein kinase C (PKC)- and cyclic AMP-responsive element binding protein (CREB)-dependent manner, which is often elevated in diverse cancers including CaP [18, 19, 29, 30]. Since the tCCN1-Prom has AP-1 binding sites, we determined expression of c-Jun, which is a cofactor for AP-1 binding, in a panel of CaP cell lines. As compared to normal immortal prostate epithelial cell lines, RWPE-1 and P69, CaP cells including PC-3, PC-3ML, DU-145 and ARCaP-M showed higher expression of c-Jun (Fig. 1A). This observation suggested that the tCCN1-Prom could serve as a cancer-selective prom for making cancer-selective oncolytic Ads and potentially *CTVs*. To monitor promoter activity quantitatively we cloned the 5′-flanking promoter region of the tCCN1-Prom (approximately 830-bp of the full length CCN1*-*Prom), inserted it upstream of a *luciferase* reporter gene and evaluated cancer-selective activity of this tCCN1-Prom in CaP cell lines. As predicted, transfection with pGL3.tCCN1-*luc* resulted in elevated luciferase expression in CaP cells as compared to RWPE-1 or P69 cells. Moreover, in the ARCaP series of CaP cells, ARCaP-M cells derived from androgen repressed human CaP (ARCaP) cells with bone metastatic capacity *in vivo* and a mesenchymal phenotype had higher tCCN1-Prom expression as compared to its epithelial-like counterpart, ARCaP-E (Fig. 1B). CCN1-Prom activities were also elevated in a series of single-oncogene transformed Fischer rat embryo cell (CREF) lines (CREF-*src*, CREF-*raf*, CREF-*ras* and CREF-*HPV*) [20, 31-33] as compared to the immortal non-transformed parental CREF cell line [34] and activity increased with the addition of tumor-promoting phorbol ester TPA (Fig. 1C), further supporting the elevated activity of tCCN1-Prom in cancer progression and metastases. Interestingly, in metastatic cells, e.g., PC-3, PC-3ML and ARCaP-M, tCCN1-Prom activity was significantly higher than the cancer-specific PEG-Prom [35], which also shows elevated cancer-specific expression in human CaP cells (Fig. 1B). To confirm these *in vitro* observations, comparative *in vivo* promoter studies were performed in nude mice containing DU-145 xenografts by injecting 10^10^ vp of either Ad.tCCN1-*luc* or Ad.PEG-*luc* intratumorally into the left or right flank, respectively, of the same mouse. BLI signals were significantly (p=0.035) higher in Ad.tCCN1-*luc*-injected tumors as compared to Ad.PEG-*luc*-injected tumors (Fig. 1D and 1E). Based on these observations, we constructed Ad.tCCN1-*E1A-mda*-7 (Ad.tCCN1-*CTV-m*7), a conditionally replication-competent Ad (CRCA) in which the tCCN1-Prom controls the Ad replication genes *E1A* and *E1B* and a *CMV*-Prom, a ubiquitous expressing promoter, controls *mda-*7/IL-24. This was accomplished using two shuttle vectors, pE1.2 and pE3.1-CMV, as shown in Suppl. Fig. 1.

As shown in Suppl. Fig. 2A, enhanced expression of MDA-7/IL-24 was evident in Ad.tCCN1-*CTV*-*m*7-infected CaP cells as compared to a normal immortal prostate epithelial cell line, RWPE-1. In contrast, when using a constitutive non-cancer specific promoter, such as CMV, expression of the transgene (in this case *mda-*7/IL-24) was evident in RWPE-1 cells, even when infected at low MOI (Supp. Fig. 2A). At a higher MOI (i.e., 1,000 vp/cell), MDA-7/IL-24 expression was lower in Ad.tCCN1-*CTV*-*m*7-treated CaP cells (Suppl. Fig. 2A), possibly due to pronounced oncolysis. Since MDA-7/IL-24 is a secretory cytokine, we quantified MDA-7/IL-24 protein levels in the conditioned medium (CM) by ELISA. MDA-7/IL-24 protein in the CM of Ad.tCCN1-*CTV-m*7-treated DU-145 and PC-3 cells was significantly higher (p<0.001) as compared to non-replicating Ad.*mda*-7-treated DU-145 and PC-3 cells infected with similar MOI (100 vp/cell) (Suppl. Fig. 2B). Additionally, the expression of secretory MDA-7/IL-24 protein in the CM of RWPE-1 did not show any significant changes between Ad.tCCN1-*CTV-m*7 vs. Ad.*mda-*7-infected groups, indicating diminished tCCN1-Prom activity leading to diminished Ad replication and MDA-7/IL-24 secretion in normal epithelial prostate cells.

Previously, we showed that Ad.PEG-*CTV-m*7 reduced tumor burden at the injection site as well as decreased tumor size at a distant location [10-12, 22] using *in vivo* xenograft models. In order to determine whether increased promoter activity of tCCN1 as compared to PEG-3 would enhance cancer-selective activity by reducing tumor burden in CaP, DU-145-*luc* cells were injected s.c. in both flanks of male nude mice. When the tumors reached ~100 mm^3^ in size with distinct BLI signals, mice were randomly divided into four groups that received (i) Ad.tCCN1*-CTV-m*7, (ii) Ad.PEG*-CTV-m*7 (iii) Ad.*vec* (control) or (iv) Ad.tCCN1-*E1A* intratumorally (i.t.) only in the right flank as indicated in Materials and Methods. Although in the treatment protocol both *CTV*s diminished tumor burden in the primary and secondary site (un-injected tumor) as compared to control groups, Ad.tCCN1*-CTV-m*7 was more potent at a similar MOI than Ad.PEG*-CTV-m*7 in reducing tumor size as measured by BLI, especially in the un-injected secondary tumor as observed after 28 days of treatment (Fig. 1F and 1G). Moreover, we observed that both Ad.tCCN1*-CTV-m*7 and Ad.tCCN1-*E1A* (CRCA) reduced the size of the primary tumor (injected tumor), but the reduction of the secondary tumor was more prominent with Ad.tCCN1-*CTV-m*7 and Ad.PEG-*CTV-m*7 as compared to Ad.tCCN1-*E1A* (CRCA lacking *mda*-7/IL-24) infected mice, supporting the superiority of a CRCA with an additional therapeutic arm, such as *mda*-7/IL-24, e.g., Ad.tCCN1-*CTV-m*7 and Ad.PEG-*CTV-m*7 (our previously generated *CTV*), in diminishing tumor growth at distant sites.

### Dose-dependent growth inhibition of CaP cells by Ad.tCCN1-*CTV-m*7

**Figure 2:**
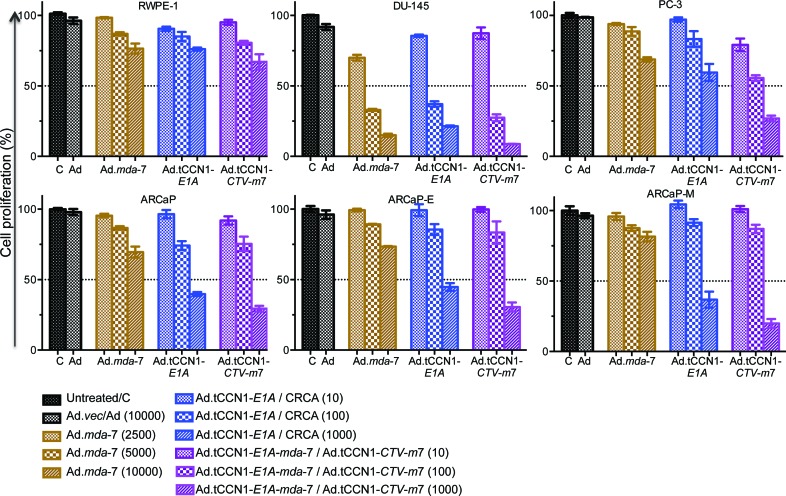
Selective inhibition of CaP growth following infection with Ad*.mda*-7, Ad.tCCN1-*E1A* (CRCA) and Ad.tCCN1-*CTV-m*7 MTT assays performed at 72-h were used to determine the effect of various concentrations (vp) of Ad.vec, Ad.*mda-*7, Ad.CCN1-*E1A* and Ad.tCCN1-*CTV-m*7 on growth of normal immortal RWPE-1 prostate cells and CaP cells, DU-145, PC-3, ARCaP, ARCaP-E and ARCaP-M cells. Results are the average + S.D. from 3 replicate samples.

To test efficacy and selectivity of an Ad.tCCN1-E1A (CRCA) and Ad.tCCN1-*CTV-m*7, we infected a panel of CaP cell lines with Ad.*vec* (Ad) (10,000 vp/cell), Ad.tCCN1-*E1A* (CRCA) (10-1,000 vp/cell), replication incompetent Ad.*mda-*7 (2,500-10,000 vp/cell) and Ad.tCCN1-*E1A-mda-*7 (Ad.tCCN1-*CTV-m*7) (10-1,000 vp/cell) for 72 h. As anticipated, the Ad.tCCN1-*CTV*-*m*7 was more efficient in growth inhibition of CaP cells than either Ad.*vec* or replication incompetent Ad.*mda-*7 (Fig. 2). Both Ad.tCCN1-*E1A* (CRCA) and Ad.tCCN1-*E1A-mda-7* (Ad.tCCN1-*CTV-m*7) induced selective growth inhibition in CaP cells in a dose-dependent manner. Results from *in vitro* studies suggest preferential killing through oncolysis promoted by the tCCN1-CRCA and *CTV-m*7, rather than by the ‘*bystander*’ effects produced by secreted MDA-7/IL-24. A significant difference (p<0.05) was evident between Ad.*vec*-infected and Ad.tCCN1-CRCA- (Ad.tCCN1-*E1A)* and Ad.tCCN1-*CTV-m*7-infected CaP cells, even with as little as 100 vp/cell. However, no significant difference was apparent between Ad.*vec*-, Ad.tCCN1-CRCA*-* or Ad.tCCN1-*CTV*-infected RWPE-1 cells at lower MOI (100 vp/cell), supporting the cancer-selective properties of these conditionally oncolytic viruses.

### Apogossypolone derivative BH3 mimetics sensitize CaP to *mda*-7/IL-24-mediated killing

**Figure 3:**
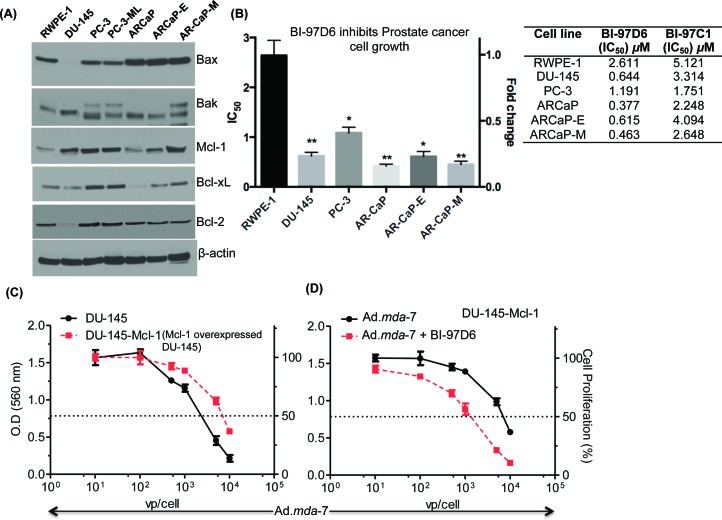
Apogossypolone derivative BH3 mimetic, BI-97D6, sensitizes CaP to *mda*-7/IL-24-mediated killing A. Western blotting of whole cell lysates of Bcl-2 family of proteins, Bax, Bak, Mcl-1, and Bcl-xL. β-actin was used as a loading control. B. IC_50_ values of CaP cell lines treated with various concentrations of the Apogosypolone derivative BI-97D6 for 72-h. CaP cells, DU-145, PC-3, ARCaP, ARCaP-E, and ARCaP-M, and normal cells, RWPE-1, were treated with various concentrations of BI-97D6 for 72-h followed by MTT assays. IC_50_ was calculated using GraphPad PRISM 5.0. The measurements were performed in triplicates. Column (Average) and Error bar (S.D). * (p<0.05), ** (p<0.01) indicates the statistical significance by using unpaired t-test between treated normal vs. treated cancer cells. C. DU-145 and Mcl-1 overexpressing DU-145 clones (DU-145-Mcl-1) were treated with increasing doses of Ad.*mda*-7, and proliferation was determined by MTT assays 3 days post-infection. D. DU-145-Mcl-1 cells were treated with Ad.*mda*-7 and or BI-97D6 for 3 days and MTT assays were performed.

CaP tumor samples from patients often overexpress specific Bcl-2 family member proteins, especially Mcl-1 and Bcl-xL [36-38]. Accordingly, expression of anti-apoptotic proteins, Bcl-2, Mcl-1, Bcl-xL, and apoptotic proteins, Bax and Bak, in CaP and RWPE-1 cells was determined using Western blotting analysis (Fig. 3A). As shown in Fig. 3A, both Mcl-1 and Bcl-xL were elevated in CaP cells as compared to RWPE-1 cells. These Bcl-2 family proteins contain hydrophobic clefts as studied by X-ray crystallography and nuclear magnetic resonance (NMR) that are able to bind the BH3 dimerization domain of pro-apoptotic molecules. This strategy led to the development of a series of BH3 mimetics or pan-Bcl-2 inhibitors, including Apogossypol, Apogossypolone and derivatives [28] as shown in Suppl. Fig. 3. To examine the apoptotic potential of Apogossypol and Apogossypolone derivatives, we treated CaP cells, including DU-145, PC-3, ARCaP, ARCaP-E, ARCaP-M, and normal immortal prostate epithelial cells, RWPE-1, with BI-97C1 or BI-97D6 [28] and found that both compounds inhibited cell growth in a dose-dependent manner. The IC_50_ values of BI-97C1 and BI-97D6 in inhibiting cell growth of DU-145, PC-3, ARCaP, ARCaP-E, ARCaP-M and RWPE-1 are shown in Fig. 3B and Suppl. Fig. 4. The optically active Apogossypolone derivative BI-97D6 was more potent in inducing CaP-cell killing than BI-97C1. Additionally, the IC_50_ value of BI-97D6 for CaP cells was significantly different vs. RWPE-1.

In previous studies, we demonstrated that suppression of Bcl-2 family member proteins is necessary to promote the growth suppressing potential of *mda-*7/IL-24 [23, 38]. Interestingly, forced expression of Mcl-1 reduced *mda-*7*/*IL*-*24-mediated apoptosis in CaP cells, which was reversed upon combined treatment with BI-97D6 and *mda-*7/IL-24 (Fig. 3C and 3D) [20, 39, 40]. Considering these observations, we hypothesized that combining a BH3 mimetic, such as BI-96C1 or BI-97D6, with Ad.*mda*-7 would inhibit CaP cell growth and viability and the combinatorial effect would be enhanced further with Ad.tCCN1-*CTV-m*7 as compared to the replication incompetent Ad.*mda*-7, specifically at a lower MOI.

### BI-97D6 and *mda*-7/IL-24 cooperate to induce apoptosis by enhancing ER stress-regulated protein expression and translation of *mda*-7/IL-24 mRNA into protein

**Figure 4:**
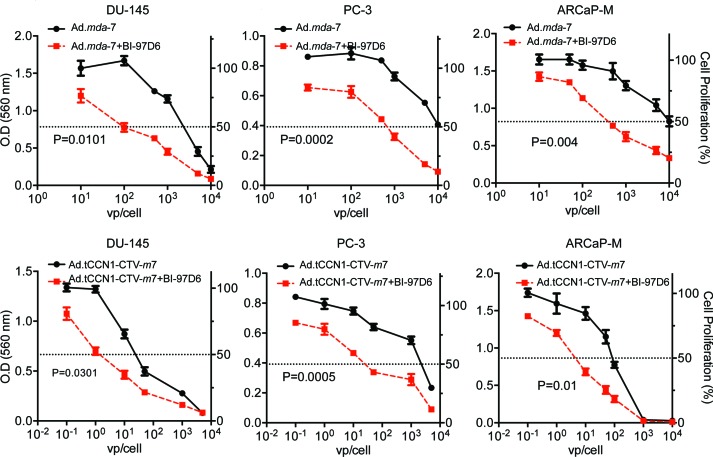
BI-97D6 potentiates *mda*-7/IL-24-induced inhibition of CaP cell growth *in vitro* DU-145, PC-3 and ARCaP-M cells were infected with the indicated M.O.I. (vp) of Ads followed by treatment with a sub-lethal dose of 250 nm BI-97D6. Cell proliferation was assessed after 72-h using MTT assays.

To investigate the potential combinatorial effect of BI-97D6 and *mda*-7/IL-24 on cell growth, CaP cells were infected with either Ad.*mda*-7 or Ad.tCCN1-*CTV-m*7 followed by treatment with a sub-lethal dose of BI-97D6 (250 nM) and MTT assays were performed. Cell growth was significantly decreased in DU-145, PC-3 and ARCaP-M cells treated with Ad.*mda*-7 plus BI-97D6, as compared to Ad.*mda*-7 treatment alone (Fig. 4). Similarly, combining Ad.tCCN1-*CTV-m*7 with BI-97D6 enhanced the decrease in cell growth as compared to cells treated singly with Ad.tCCN1-*CTV-m*7. As compared to the non-replicating Ad.*mda-*7, a conditionally replicating Ad.tCCN1-*CTV-m*7 was superior in inhibiting CaP cell growth. The IC_50_ of Ad.tCCN1-*CTV-m*7-infected CaP cells was ~100-fold lower as compared to the non-replicating Ad.*mda*-7. This is clinically relevant, since administering a sufficient titer of *CTV-m*7 can easily be achieved based on its cancer-selective replication at the tumor site, which is not achievable with a non-replicating Ad.*mda*-7, and activity can be further potentiated by using BH3 mimetics, e.g., BI-97D6.

**Figure 5:**
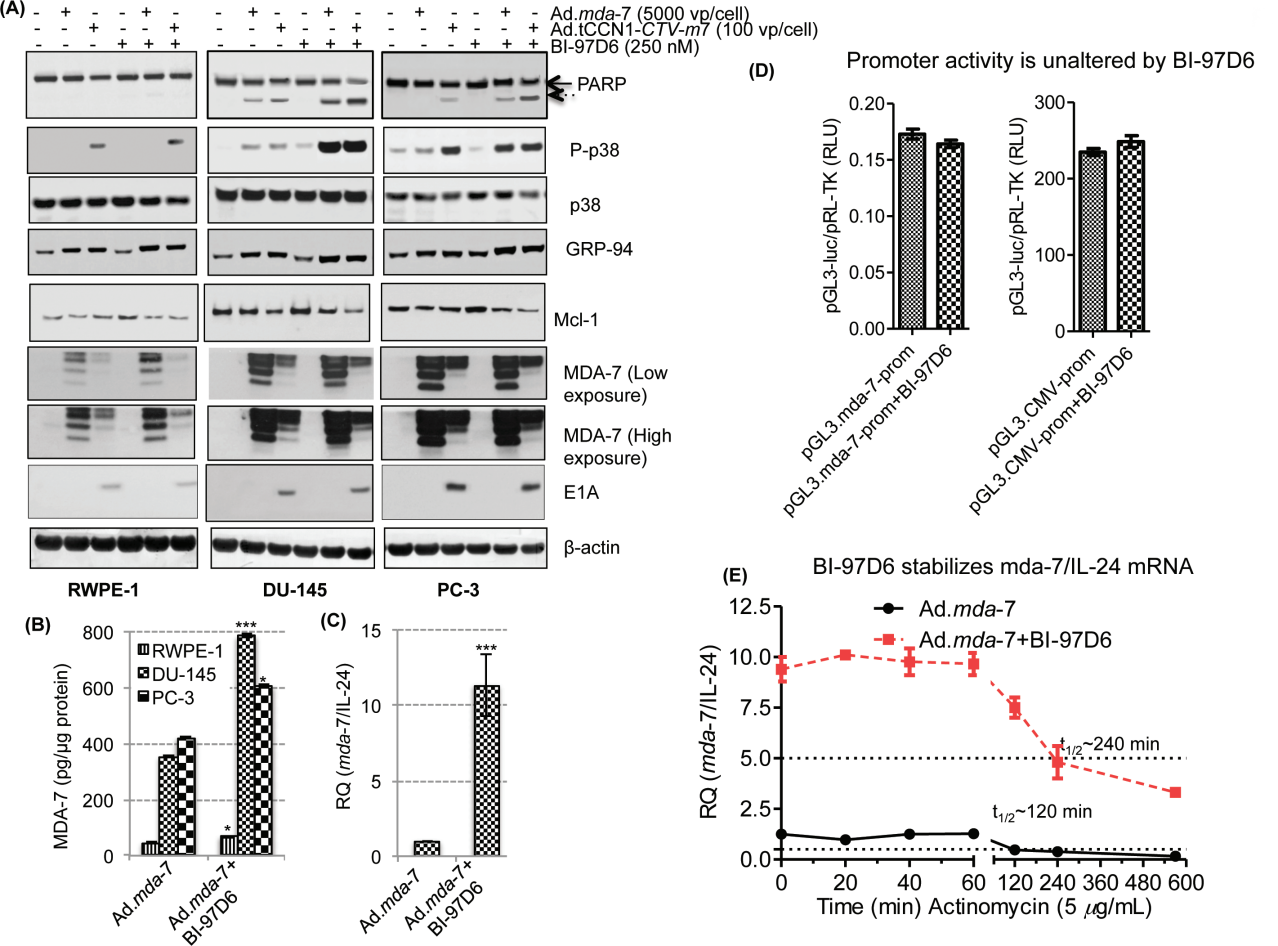
BI-97D6 potentiates *mda*-7/IL-24-induced apoptosis by enhancing ER stress-regulated protein expression as well as enhancing the translation of *mda*-7/IL-24 mRNA DU-145, PC-3 and normal RWPE-1 cells were infected with 5,000 vp/cell of Ad.*mda*-7 or 100 vp/cell of Ad.tCCN1-*E1A-mda*-7 (Ad.tCCN1-*CTV-m*7) followed by BI-97D6 (250 nM) treatment. Cell lysates were prepared 48-h post infection and Western blotting was performed. A. B. Quantification of MDA-7/IL-24 protein in Ad.*mda*-7 and Ad.*mda*-7 + BI-97D6 cells using a human IL-24 Elisa Kit. ***(p<0.001) indicates statistical significance between the indicated experimental groups. C. q-PCR of DU-145 cells treated with Ad.*mda-*7 alone or in combination with BI-97D6. GAPDH was used as a loading control for relative quantification of *mda*-7/IL-24 mRNA. D. RLU of *mda*-7-Prom (pGL3.*mda*-7-Prom-*luc*) and *CMV*-Prom (pGL3.*CMV*-Prom-*luc*) after normalization with p*RL*-TK. E. BI-97D6 enhances the stability of mda-7/IL-24 mRNA. DU-145 cells were treated with Ad.*mda*-7 and/or BI-97D6 for 24 h followed by treatment with Actinomycin D (5 μg/ml). Cells were collected at indicated time points post-treatment and qPCR was performed.

Experiments were next performed to interrogate the potential molecular mechanism by which the combination of *mda-*7/IL-24 and BI-97D6 affects the growth and survival of CaP cells. We observed an increase in phosphorylation of p38 as well as the ER stress marker GRP-94 following Ad.*mda*-7 or Ad.CCN1-*CTV-m*7 infection and a decrease in expression of the anti-apoptotic Mcl-1 protein. MDA-7/IL-24 expression in cell lysates was lower in Ad.tCCN1-*CTV-m*7 as compared to Ad.*mda*-7 infected cells at 48 h, which was partially due to the lower MOI (100 vp/cell) of Ad.tCCN1-*CTV-m*7 used as compared to the higher MOI (5,000 vp/cell) of Ad.*mda*-7, and also a consequence of the pronounced oncolytic effect that diminished cell survival and protein production. However, both infective doses promoted similar levels of induction of ER stress proteins, i.e., p38 and GRP-94, which was enhanced further when viruses were combined with BI-97D6 leading ultimately to apoptosis (Fig. 5A). Apoptotic death was the preferred mode of cell killing in DU-145 cells as evidenced by PARP cleavage (Fig. 5A), whereas minimum cell death was observed in RWPE-1 cells. PARP cleavage was not as prominent in PC-3 as compared to DU-145 using a similar MOI of Ads. This may be due to intrinsic therapy- and apoptosis-resistance of PC-3 as compared to DU-145. Moreover, PC-3 expresses reduced CAR resulting in restricted entry of Ad and consequently lower transgene expression [41, 42], but interestingly the apoptosis-inducing combinatorial effect of Ad.tCCN1-*CTV-m*7 and BI-97D6 was also evident in PC-3 as compared to either agent alone.

Interestingly, as predicted, the level of secreted MDA-7/IL-24 protein in CM of normal RWPE-1 cells infected with Ad.tCCN1-*CTV-m*7 was extremely low as compared to both DU-145 and PC-3 (Suppl. Fig 5), reflecting the cancer-selective replication of Ad.tCCN1-*CTV-m*7 with concomitant production of MDA-7/IL-24 protein. The selective replication of Ad.tCCN1-*CTV-m*7 in CaP cells was also confirmed by the detection of *E1A* expression in infected DU-145 and PC-3 as compared to RWPE-1 (Fig. 5A). The cancer-selective apoptosis by *mda*-7/IL-24 along with the cancer-selective oncolytic effects of Ad.tCCN1-*CTV-m7* were more pronounced after a longer incubation, as the *CTV*s were able to replicate exponentially with time thereby producing elevated levels of secreted MDA*-*7/IL-24 protein (Suppl. Fig. 5).

MDA-7/IL-24 levels were measured with a h-IL-24 ELISA kit using several fold dilution of the lysates as well as conditioned medium to define the relative amounts of MDA-7/IL-24 in the detection range of the kit. The level of expression of MDA-7/IL-24 was enhanced by treatment with BI-97D6 when cells were treated with Ad.*mda*-7 plus BI-97D6 vs. Ad.*mda*-7 alone (Fig. 5B). As shown in Fig. 5C, BI-97D6 also enhanced *mda-*7/IL-24 mRNA levels. Similar results were obtained with another class of BH3 mimetics, BI-97C1 (Sabutoclax), suggesting that this phenomena may be related to inhibition of Mcl-1 expression (Suppl. Fig. 6). To determine if transcription of the *mda*-7/IL-24 gene changed following Mcl-1 inhibition, we tested promoter activity of the *CMV*-Prom and *mda*-7-Prom following BI-97D6 treatment and found that the regulation of *mda*-7/IL-24 was not altered at a transcriptional level (Fig. 5D). This prompted us to investigate the level of MDA-7/IL-24 at a post-transcriptional level. DU-145 cells were treated with Ad.*mda*-7 and/or BI-97D6 for 24 h followed by addition of Actinomycin D (5 μg/ml) to stop new mRNA transcription and mRNA stability was monitored. The cells were collected at different time points post-treatment with Actinomycin D, mRNA was isolated and qPCR was performed using an *mda*-7/IL-24 probe, and a GAPDH probe was used as loading control. The half-life (t_1/2_) of *mda*-7/IL-24 mRNA was ~120 min, which was increased to ~240 min by the addition of BI-97D6 (Fig. 5E). These results suggest that BI-97D6, which inhibits Mcl-1 expression, enhances the steady state level of *mda*-7/IL-24 mRNA.

### The combination of BI-97D6 and Ad.tCCN1-*CTV-m7* decreases prostate tumor size in transgenic prostate cancer Hi-*myc* mice

**Figure 6:**
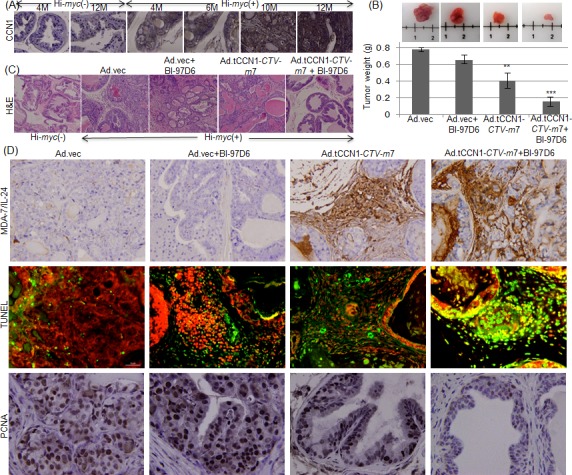
Ad.tCCN1-*CTV-m7* in combination with BI-97D6 significantly reduces prostate tumor development in Hi-*myc* prostate cancer transgenic mice A. IHC of CCN1 expression in Hi-*myc*(−) and Hi-*myc*(+) mice of matched ages. Magnification: 10X B. 5-6 month old Hi-myc(+) mice were randomly divided into 4 groups (n = 5): 1) Ad.vec, 2) Ad.vec + BI-97D6, 3) Ad.tCCN1-*CTV-m*7 and 4) Ad.tCCN1-*CTV-m*7 + BI-97D6. Mice with prostate tumors were injected through their tail veins with the indicated complement-treated Ad/D-MB complex and, 6 min post-injection, were sonoporated in the prostate region for 10 minutes. Mice were treated for 4 weeks (2 injections/week). BI-97D6 was administered i.p for 4 weeks (3 injections/week) at a dose of 3 mg/kg body weight. Mice were sacrificed and tumors were photographed and weighed (shown in grams). Graph depicts tumor weights of the treatment groups. C. H&E staining of representative treated groups of Hi-*myc*(+) mice. Magnification; 100X. D. IHC of MDA-7/IL-24 and PCNA expression was done on formalin-fixed paraffin-embedded tumor sections from treated mice. Apoptosis was measured by TUNEL staining. Magnification; 400X.

To fully appreciate the totality of Ad.tCCN1-*CTV-m7* properties as a gene therapeutic vector, which include direct oncolysis, anti-tumor MDA-7/IL-24 effects via direct cancer cell apoptosis and through potent ‘*bystander antitumor effects*’ [10, 15, 16, 43], as well as involvement of components of the immune system [44, 45], it is essential to validate efficacy in an immune-competent CaP animal model, such as the Hi-*myc* mouse [46]. Hi-*myc* transgenic mice share molecular characteristics of human CaP. They develop prostatic intraepithelial neoplasia (mPIN) as early as 2 weeks and invasive prostate adenocarcinoma by 6 months of age [46]. Interestingly, CCN1 protein becomes elevated in 6-month old Hi-*myc* mice as they develop locally invasive prostate cancer (Fig. 6A). Expression of CCN1 is significantly elevated in Hi-*myc*(+) mice vs. age-matched groups of normal or Hi-*myc*(−) mice, suggesting the potential utility of the cancer-selective tCCN1-Prom for developing oncolytic viruses with concomitant production of a therapeutic cytokine, a *CTV-m7* in this model.

The UTMD approach [22, 23] was used with targeted/decorated MBs (D-MBs), complex, Biotin-anti-V-CAM-1-Streptavidin-MBs (MB-SA-B-anti-VCAM-1; D-MBs), to deliver Ad.tCCN1-*CTV-m7* as described in Materials and Methods. D-MBs were treated with complement prior to injection into the tail vein of Hi-*myc* mice, which partially shielded the Ad.tCCN1-*CTV-m7* in the circulation, avoiding direct activation of the immune system [47-49]. The viral particles were then released at the target site by sonoporation using ultrasound [22, 23].

To investigate the antitumor effects of Ad.tCCN1-*CTV-m7*, alone or in combination with BI-97D6, 5-6 month old Hi-*myc* mice were divided into 4 groups (n=5): i) Ad.vec; ii) Ad.vec + BI-97D6; iii) Ad.tCCN1-*CTV-m*7; and iv) Ad.tCCN1-*CTV-m*7 + BI-97D6. Ad.vec or Ad.tCCN1-*CTV-m7* were mixed with D-MBs and the resultant complexes, i.e., D-MB/*CTV-m*7 or D-MB/Ad.vec, were systemically administered via tail vein injection and sonoporated as described in Materials and Methods. BI-97D6 was i.p. injected (3X per week for a period of 4 weeks) at a dose of 3mg/kg mouse body weight. The experiment was terminated after 4 weeks and mice were sacrificed and the prostate tumors were collected. There was a significant decrease in tumor size in the Ad.tCCN1-*CTV-m7*-treated group as compared to the Ad.vec-treated group (Fig. 6B). Tumors were weighed and showed a significant decrease in tumor weight (p<0.01) in the Ad.tCCN1-*CTV-m*7-treated group as compared to Ad.vec-treated group with a further decrease in tumor weight (p<0.001) in the Ad.tCCN-*CTV-m7* + BI-97D6-treated group (Fig. 6B). H&E staining of sections prepared from the treated mice further confirmed the efficacy of the single and combination treatments. Invasive prostatic adenocarcinomas were observed in Ad.vec-treated 6-month old Hi-*myc* mice, whereas the invasive prostatic adenocarcinomas displayed decreased size in the Ad.tCCN1-*CTV-m7*-treated group and were barely detectable in the combination-treated groups. The prostate glands of 6-month old Hi-*myc* mice receiving the combination of Ad.tCCN1-*CTV-m*7 + BI-97D6 appeared normal in comparison with matched age group wild type or Hi-*myc* (−) mice (Fig. 6C) with minimal symptoms of prostatic intraepithelial neoplasia (PIN). These results indicate that prostate pathology observed in Hi-myc mice can be inhibited by treatment with Ad.tCCN1-*CTV-m*7+ BI-97D6, suggesting potential clinical significance of this combination in treating CaP.

The decrease in tumor size was associated with a reduction in cell proliferation as evidenced by lack of PCNA expression and increased cell death reflected by positive TUNEL staining (Fig. 6D). The expression of MDA-7/IL-24 in the prostate gland was also evident in Ad.tCCN1-*CTV-m*7- and combination-treated animals (Fig. 6D). Although, there was cell death and inhibition of cell proliferation in the single-treated group (i.e., Ad.*vec* + BI-97D6 or Ad.CCN1-*CTV-m*7), the effect was more pronounced and robust in the combination-treated group (Fig. 6D).

## DISCUSSION

Conditional replication-competent Ad (CRCA) and oncolytic viruses engineered to propagate and lyse tumor cells have been used in various clinical trials and were safe when delivered intratumorally in various Phase II/III clinical trials [50]. However, the efficacy of these viruses in treating various cancers has been limited [13, 14, 51, 52]. In order to increase the selectivity, efficacy and safety of viral CaP therapy, organ-specific promoters targeting prostate epithelial cells have been utilized to drive prostate-specific replication competent Ads, e.g., *CV706* or Ad(I/PPT-*E1A*) or *CG7870* [5, 53, 54], which appeared safe and promoted a tumor response when injected intra-prostatically [5, 53, 54]. Although organ- or tissue-based promoters may be effective in the treatment of primary tumors when delivered directly into the tumor, efficacy is compromised when they are administered systemically to treat primary tumors and metastatic disease [13, 14, 23].

To enhance the cancer therapeutic efficacy of CRCAs, particularly in the context of metastatic disease, we developed unique bipartite CRCAs displaying cancer-specific replication and simultaneous production of a therapeutic cytokine [11-14, 17, 49]. This was accomplished by using a ubiquitous cancer-selective minimal promoter derived from rodent progression elevated gene-3 (*PEG*-3) [35] to control *E1A* expression, with concomitant production of the IL-10 gene family member *mda*-7/IL-24 that induces cancer-selective apoptosis and toxic autophagy [45, 55], referred to as a Cancer Terminator Virus (*CTV*; Ad.PEG-*E1A-mda*-7; Ad.PEG-*CTV-m*7) [10-14, 22]. Previous studies indicated that the *CCN1/CYR61* gene and its promoter are upregulated in various cancers and responsible for malignant and metastatic transformation [19, 20]. Clinical studies suggest that the expression of CCN1 correlates with tumor stage, size, lymph node involvement, and represents a poor prognostic factor in various cancers, including prostate [18, 19, 56]. Based on these considerations, we determined the activity of a truncated tCCN1-Prom in a collection of CaP cells. As predicted, the tCCN1-Prom was upregulated in all CaP cells in comparison to RWPE-1 normal prostate epithelial cells. Moreover, the activity of tCCN1-Prom was more prominent in metastatic CaP cell lines, e.g., PC-3, PC-3ML and ARCaP-M, as compared to less aggressive CaP cells. Additionally, the tCCN1-Prom had higher activity (p<0.05) as compared to the PEG-Prom in CaP metastatic cells, supporting the potential utility of the tCCN1-Prom in generating engineered CRCAs and *CTVs*. In this report, we have generated a *CTV* in which the tCCN1-Prom drives replication with simultaneous production of *mda*-7/IL-24, e.g., Ad.tCCN1-*E1A*-*mda*-7 (Ad.tCCN1-*CTV-m*7). Ad.tCCN1-*CTV-m*7 displays enhanced activity in CaP both *in vitro* and *in vivo* as compared to Ad.PEG-*CTV-m*7 and Ad.*mda-*7 [10, 22, 49] (Figs.1 and 2).

The inability to efficiently and selectively administer CRCAs systemically has restricted their ability to successfully treat patients with cancer, including primary tumors and metastases [13, 14, 22, 47]. To mitigate these hurdles we are using complement-treated polycarbonate microbubbles (MBs) and ultrasound, i.e., ultrasound targeted microbubble destruction (UTMD), to reduce trapping of viruses non-specifically in organs such as the liver and to diminish immune detection and clearance [12, 22, 23]. The UTMD approach has been used to selectively deliver in a “stealth manner” both non-replicating viruses and *CTVs* systemically to tumors in both nude mice containing human CaP xenografts and immune competent transgenic animals developing CaP, resulting in significant pre-clinical anti-cancer activity [22, 23]. To refine further the UTMD approach, we have now used modified (decorated) microbubbles (D-MBs) in which biotinylated anti-V-CAM-1 is complexed with streptavidin microbubbles (MB-SA), resulting in D-MBs, i.e., biotin-anti-V-CAM-1-streptavidin-MBs. These D-MBs accumulate in the tumor vasculature and after US the contents of the MBs, which contain either Ad.vec or Ad.tCCN1-*CTV-m*7, are released and can now infect prostate cells in the Hi-*myc* mice (Fig. 6). This strategy has wide applicability for delivering therapeutic agents (including viruses, therapeutic nucleic acids, drugs) to tumor cells and when combined with the ability to produce and secrete a therapeutic cytokine (such as *mda-*7/IL-24 in the *CTV*) engenders this approach with the ability to treat both primary and metastatic tumors.

The effectiveness of CRCAs as cancer therapeutics in both pre-clinical and clinical settings is enhanced when used in combination with other therapeutic modalities, including chemotherapy, monoclonal antibody therapy and radiation therapy [57, 58]. A critical determinant of combinatorial efficacy is the choice of the most appropriate therapeutic agent to use with the CRCA for a specific cancer indication. The Bcl-2 family of proteins, especially Bcl-xL and Mcl-1, were found to be elevated in CaP and higher expression correlated with therapy resistance, thus representing a potential target for primary cancer treatment and combinatorial treatment with CRCAs [36-38 59]. Bcl-2 family proteins contain a hydrophobic cleft that binds with only BH3-motif containing pro-apoptotic Bcl-2 family proteins (Bax, Bak, Bad), antagonizing apoptosis and enhancing cell survival. Structural analyses using X-ray crystallography and NMR assisted in designing various chemically synthesized BH3 mimetics that inhibit the binding of BH3-containing pro-apoptotic proteins with anti-apoptotic Bcl-2 proteins, thereby inducing apoptosis via a Bcl-2-dependent pathway.

We demonstrated previously that *mda*-7/IL-24-induced ER-stress response caused apoptosis in CaP cells by inhibiting Mcl-1 translation, which was reversed by overexpression of Mcl-1 [38]. These observations suggested that BH3 mimetic Mcl-1 inhibitors and *mda-*7/IL-24 might cooperate synergistically to induce apoptosis in CaP. This hypothesis has been validated by the findings that the Apogossypol derivative BI-97C1 (Sabutoclax), which targets Mcl-1, sensitized CaP cells to *mda*-7/IL-24-mediated toxicity [23]. The Apogossypolone derivative BI-97D6 is potentially ~5-10-fold more active than the Agogossypol derivative BI-97C1 (Sabutoclax) in inhibiting the binding of BH-3 peptides to Bcl-xL, Bcl-2, Mcl-1 and Bfl-1 [28]. In this context, we hypothesized that BI-97D6 might prove more potent than BI-97C1 (Sabutoclax) in abrogating the activities of the protective Bcl-2 family of proteins especially Mcl-1. Indeed we showed that BI-97D6 selectively induced CaP cell death (Fig. 3), reversed therapy-resistance and enhanced *mda*-7/IL-24-mediated cell killing (Fig. 4). Additionally, we showed that BI-97D6 and Ad.tCCN1-*CTV-m*7 cooperatively induced ER stress-mediated apoptosis. Interestingly, our observations indicated that BI-97D6 also stabilized *mda-*7/IL-24 mRNA, thus enhancing the production of MDA-7/IL-24 protein and concurrently *mda-*7/IL-24-mediated apoptosis (Fig. 5). *mda*-7/IL-24 contains a 3′UTR with AU-rich elements (ARE, which lead to unstable *mda-*7/IL-24 mRNA [60]). *mda*-7/IL-24-inducers regulate the expression of this transgene at the post-transcriptional level by stabilizing its mRNA [60, 61]. These previous findings are compatible with our current results, which establish BI-97D6 as an inducer of *mda*-7/IL-24. Our data also supports the use of Ad.tCCN1-*CTV-m*7 in complex with decorated microbubbles using the UTMD approach to effectively target the prostate gland in immunocompetent Hi-*myc* mice. This was confirmed by expression of MDA-7/IL-24 followed by cell death in CaP sections from the Hi-Myc mice that lead to diminished prostate tumor size, all of which were further enhanced by BI-97D6 (Fig. 6).

In summary, the significant CaP antitumor effects observed following combination treatment with Ad.tCCN1-*CTV-m*7 + BI-97D6 is a summation of multiple independent biological attributes of these agents including: a) cancer-selective oncolytic activity mediated by Ad.tCCN1-*CTV-m*7; b) cancer-selective antitumor effects mediated by both Ad.tCCN1-*CTV-m*7 and BI-97D6; and c) ‘*bystander effects*’ mediated by Ad.tCCN1-*CTV-m*7 through production and the secretion of MDA-7/IL-24 protein. Considering the safety profile and initial promising results of BH3 mimetics and oncolytic Ads when combined with standard chemotherapeutic regimens, defining the correct combination of agents will be pivotal to enhancing anti-tumor effects in the clinic. In principle, a combination of a BH3 mimetic, such as BI-97D6 with more pronounced Bcl-2/Mcl-1 inhibitory activity, with a CRCA such as Ad.tCCN1-*CTV-m*7 expressing a systemically active cytokine, *mda-*7/IL-24 [62-64], should elicit profound efficacy toward both primary and metastatic tumors in the clinic. Of particular clinical relevance is our demonstration that decorated (targeted) MBs can be used to encapsulate Ad.tCCN1*-CTV-m*7 and deliver this virus systemically in animals resulting in profound anti-cancer activity in Hi-myc mice, which is enhanced further when animals also receive i.p. injections of BI-97D6 (Fig. 6). Future studies are required to determine the clinical utility of this combination and the UTMD approach as a potential therapy for patients with advanced CaP.

## MATERIALS AND METHODS

### Cell culture

DU-145, PC-3 and PC-3-ML cell lines were obtained from the ATCC, maintained in EMEM, F-12K (ATCC) and RPMI (GIBCO®, Invitrogen™, Auckland, NZ) medium supplemented with 10% Fetal Bovine Serum (FBS) (Sigma-Aldrich, St. Louis, MO, USA), respectively. ARCaP, ARCaP-E and ARCaP-M cell lines were obtained from Novicure Biotechnology, maintained in MCaP medium (Novicure Biotechnology, Birmingham, AL, USA) supplemented with 5% FBS, and immortalized normal human prostate epithelial cells RWPE-1 were obtained from ATCC, maintained in Keratinocyte serum free medium (K-SFM) (Gibco) supplemented with 0.05 mg/ml BPE and 5 ng/ml EGF. Stable clones of DU-145 expressing luciferase and elevated Mcl-1 were obtained by transfecting DU-145 with pGL4.5[*luc*2/CMV/hygro] (Promega, Madison, WI, USA) and pcDNA3.1[*Mcl-1*/CMV/neo], respectively, supplemented with antibiotics hygromycin and neomycin, respectively. An immortal normal cloned Fischer rat embryo cell line (CREF) [34] and its single oncogene transformed clones (CREF-*src*, CREF-*raf*, CREF-*ras* and CREF-*HPV*) [20, 31-33] were grown in DMEM supplemented with 5% FBS. All cell lines were cultured at 37°C in a 5% CO_2_ and 95% air-humidified incubator.

### Cell growth assays

CaP and immortal normal prostate cells were treated with Ad.*vec*, Ad.*mda*-7, Ad.tCCN1-*E1A* or Ad.tCCN1-*CTV-m*7, without or with BI-97D6, and proliferation was determined by MTT assays [12].

### Contruction of Ad.tCCN1-*E1A*-*mda*-7 (Ad.tCCN1-*CTV-m7*)

To construct Ad.tCCN1-*E1A-mda*-7, AdenoQuick cloning system (OD260, Inc., Boise, ID, USA) was employed. This system utilizes two shuttle vectors (pE1.2 and pE3.1-CMV) in which the transgenes were inserted before being transferred into a large adenoviral plasmid (pAd) (Suppl. Fig. 1). The *E1A* region was deleted from pAd leaving the *E1B* region intact. The expression cassette in which the tCCN1-Prom drives early region *E1A* (tCCN1-*E1A*) of Ad was inserted into the multiple cloning site (MCS) of pE1.2 (i.e., pE1.2-tCCN1-*E1A*). The other expression cassette, in which C*MV*-Prom drives the expression of *mda*-7/IL-24, was inserted into the MCS of pE3.1-CMV (i.e., pE3.1-CMV-*mda*-7). In both shuttle plasmids the MCS is flanked by two sets of restriction sites. pE1.2-tCCN1-*E1A* and pE3.1-CMV-*mda*-7 were digested with Restriction enzymes (R.E) with *Alw*NI, *Bst*API, *Dra*III or *Pfl*MI. pAd was digested with *Sfi*I to generate sticky ends with deleted E1A (ΔE1) and E3 (ΔE3). The sticky ends of ΔE1 region are incompatible with each other and with those present in the ΔE3 region, but are compatible with those generated by digesting plasmid pE1.2-tCCN1-*E1A* with *Alw*NI, *Bst*API, *Dra*III or *Pfl*MI. The sticky ends of ΔE3 region are incompatible with each other and with those present in the ΔE1 region but are compatible with those generated by digesting plasmid pE3.1-CMV-*mda*-7 with *Alw*NI, *Bst*API, *Dra*III or *Pfl*MI. Upon ligation of the expression cassettes at the respective specific sites, the ligated product was transformed into *E. coli* to select the clones pAd.tCCN1-*E1A*-CMV-*mda*-7 with ampicillin (*amp*^R^ provided by pAd) and kanamycin resistance (*Kan*^R^ provided by shuttle vector). The resultant plasmid pAd.tCCN1-*E1A*-CMV-*mda*-7 was digested with *Pac*I to release viral ITRs and was transfected into HEK-293 cells to rescue the conditionally replication-competent Ad (CRCA); Ad.tCCN1-*E1A*-*mda*-7). Similar strategies were used to construct Ad.*mda*-7 and Ad.tCCN1-*E1A* constructs. The constructs were purified using CsCl gradient, titrated both by OD260-SDS (vp/ml) (Optical absorbance at 260 nm of lysed Ad using 0.1% Sodium dodecyl-sulphate solution) method and TCID50 (median or 50% tissue culture infective dose) or plaque forming methods (pfu/ml). We thank Drs. Curiel and Dmitriev (Washington University School of Medicine; St. Louis, MO, USA) for assistance in preparing and expanding various Ads.

### Cell transfection and luminescence assay

RWPE-1, P69, PCa (DU-145, PC-3, PC3-ML ARCaP, ARCaP-E, and ARCaP-M), CREF and transformed CREF (CREF-*src*, CREF-*raf,* CREF-*ras*, CREF-*HPV*) cells were plated at a density of 3 × 10^4^ cells/well in quadruplicate in 24-well cell culture plates. After 24 h, cells were transfected with pGL3.CMV-*luc*, pGL3.PEG-*luc* or pGL3.tCCN1-*luc* using Fugene® HD transfection reagent (Promega). For normalization, the indicated plasmids were co-transfected with pRL-TK at a ratio of 20:1. Cells were lysed after 48 h post-transfection and luminescence was studied using a Dual-Luciferase Reporter® assay system (Promega).

### *In vivo* comparison of promoter activity driving luciferase and bipartite oncolytic Ads in a human CaP tumor xenograft model

Athymic nude mice were injected s.c. in both flanks with 2 × 10^6^ DU-145 cells. When tumors reached ~100 mm^3^ in size, 10^10^ vp of Ad.PEG-*luc* or Ad.tCCN1-*luc* were injected i.t. (intratumorally) in the right and left flank tumors, respectively. Bioluminescence imaging (BLI) was done using an IVIS spectrum (Califer Life Sciences, Inc., Hopkinton, MA) [12] 72-h post injection.

Athymic nude mice were injected s.c. in both flanks with 2 × 10^6^ DU-145-*luc* (luciferase gene bearing DU-145 cell line). When tumors reached ~100 mm^3^ in size or visible by BLI, mice were randomly divided into four subgroups (n=5) receiving injections i.t. only in the right flank of either Ad.tCCN1-*CTV-m*7, Ad.PEG-*CTV-m*7, Ad.tCCN1-*E1A* or Ad.*vec* (control) at a dose of 10^10^ vp per tumor site twice a week for a period of 4-weeks. BLI was done once a week, and image analyses were performed using Living Image 4.3.1.

### Preparation of whole-cell lysates and Western blotting analyses

Cells were lysed in cell lysis buffer (Cell Signaling Technology, Inc., Danvers, MA, USA) and whole cell lysates were collected after centrifugation at 12,000 rpm for 15 min at 4°C [12]. For Western blotting analyses, the primary antibodies used were mouse monoclonal anti-MDA-7/IL-24 (1:2000; Gen Hunter Corporation, Nashville, TN, USA), anti-E1A (1:1000; EMD Millipore), anti-EF1α (1:5000; EMD Millipore), anti-β-actin (1:5000; Sigma-Aldrich), rabbit monoclonal anti-Bcl-xL (1:1000), anti-PARP (1:1000), anti-Bcl-2 (1:1000), anti-Mcl-1, rabbit polyclonal anti-phospho p-38 and anti-p38 (1:1000; Cell Signaling Technology). The secondary antibodies used were polyclonal goat anti-mouse IgG (1:1000; Dako, Carpinteria, CA, USA) and polyclonal swine anti-rabbit IgG (1:3000; Dako).

### Preparation of Ad-complexed with decorated or targeted MBs (Ad/D-MBs) and UTMD *in vivo* in transgenic Hi-myc mice

Biotinylated anti-V-CAM-1 (B-VCAM-1) (100 μg) (BioLegend, San Diego, CA, USA) was incubated and complexed with streptavidin microbubbles (MB-SA) (~10^9^ MBs) (Targeson Inc, San Diego, CA, USA) forming biotin-anti-V-CAM-1-streptavidin-MBs (MB-SA-B-anti-VCAM-1; D-MBs). Ads were then complexed as described previously [12] and finally dissolved in 1 ml PBS. Ad/D-MBs were treated with complement and systemically injected via tail vein and sonoporated 6 min after tail veil injection of Ad/D-MBs complexes using MicroMaxx® Ultrasound System with L25-e probe (SonoSite, Inc. Bothell, WA, USA) intermittently for 10 min, the UTMD approach [22, 65].

For this therapeutic experiment, spontaneous CaP developing Hi-*myc* male mice of 5-6 months of age were used. Hi-*myc* mice were randomly divided into 4 groups (n=5): i) Ad.*vec*; ii) Ad.*vec* + BI-97D6; iii) Ad.tCCN1-*CTV-m*7; and iv) Ad.tCCN1-*CTV-m*7 + BI-97D6. Ad.*vec* or Ad.tCCN1-*CTV-m*7 were mixed with D-MBs and the resultant complexes, i.e., D-MB/Ad.tCCN1-*CTV-m*7 or D-MB/Ad.*vec*, were systemically administered via tail vein injection (2 × week for a period of 4-weeks) and sonoporated as mentioned above in the prostate region. BI-97D6 was i.p. injected (3 × per week for a period of 4-weeks) at a dose of 3-mg/kg mouse body weight. The experiment was terminated after 4-weeks and mice were sacrificed and the prostate tumors were collected.

### Statistical analyses

Data presented as mean ± S.D. and plotted using GraphPad Prism 5. Student's t-test determined significance with p ≤ 0.05, ≤ 0.01 and ≤ 0.001 denoted by asterisks *, ** and ***, respectively, by comparing the experimental (treated) vs. control group.

## SUPPLEMENTARY MATERIAL, FIGURES


