# Supplementary material for: Therapy of prostate cancer using a novel cancer terminator virus and a small molecule BH-3 mimetic
Source: Oncotarget. 2015 Mar 12;6(13):10712–27. doi: 10.18632/oncotarget.3544 (PMC4484414; doi:10.18632/oncotarget.3544)
Supplement: Supplementary file 1 [file oncotarget-06-10712-s001.pdf]

Therapy of prostate cancer using a novel cancer terminator virus and a small molecule BH-3 mimetic

Supplementary Material

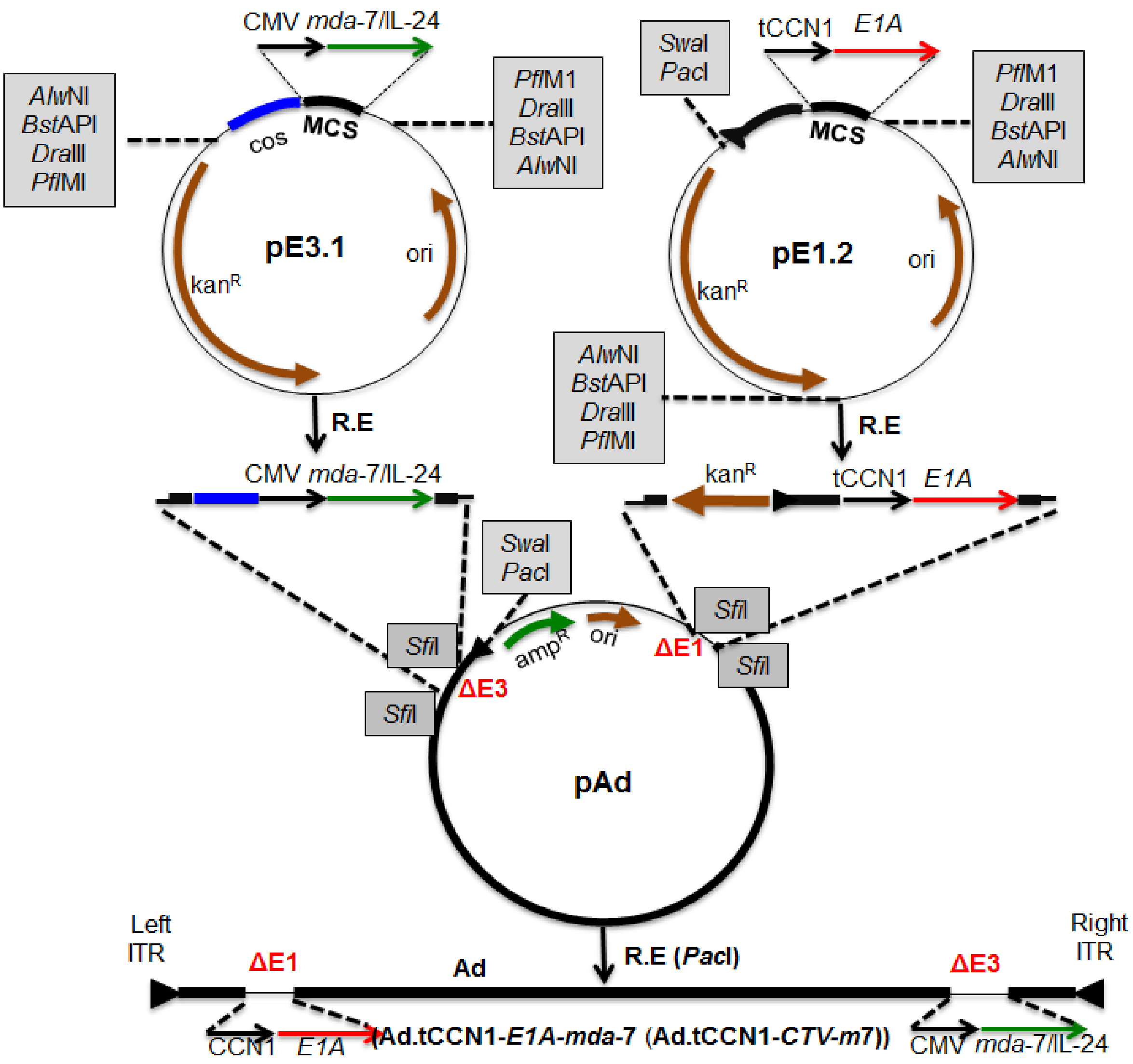

Supplementary Figure 1: Construction of Ad.tCCN1-*E1A*-*mda*-7 (Ad.tCCN1-*CTV*-*m*7). To generate Ad.tCCN1-*E1A*-*mda*-7, the AdenoQuick cloning system was employed using two-shuttle vectors pE1.2 and pE3.1 in which tCCN1-Prom driving *E1A* and CMV-Prom driving *mda*-7/IL-24 were inserted, respectively, before being transferred to large pAd as described in Materials and Methods.

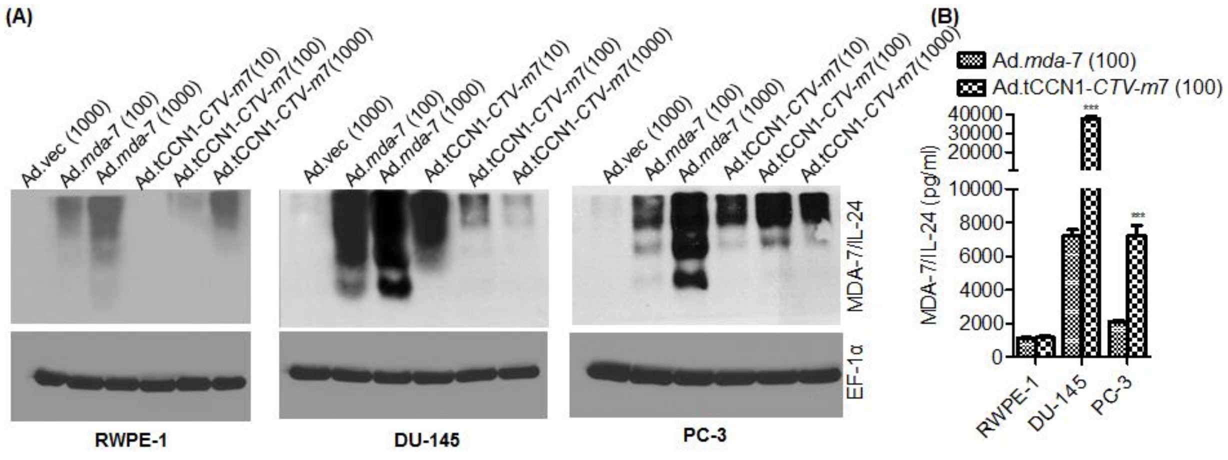

Supplementary Figure 2: Conditionally cancer-specific replication competent bipartite Cancer Terminator Virus (Ad.tCCN1-*CTV-m7*) driven by tCCN1-Prom replicates and simultaneously produces *mda*-7/IL-24 in CaP cells. *A.* MDA-7/IL-24 protein expression in CaP and RWPE-1 normal human immortalized prostate epithelial cells after infection with Ad.*vec* (1,000 vp/cell), Ad.*mda*-7 (100 and 1,000 vp/cell) and Ad.tCCN1-*CTV*-*m*7 (10, 100, 1,000 vp/cell). Cell lysates were collected after 48 h of Ad infection, and equal amounts of protein were loaded as confirmed by the loading control EF-1α. ***B.*** Relative quantification of MDA-7/IL-24 protein as measured by hIL-24 DuoSet ELISA kit in conditioned media (CM) 48 h post-infection with the indicated Ads at 100 vp/cell.

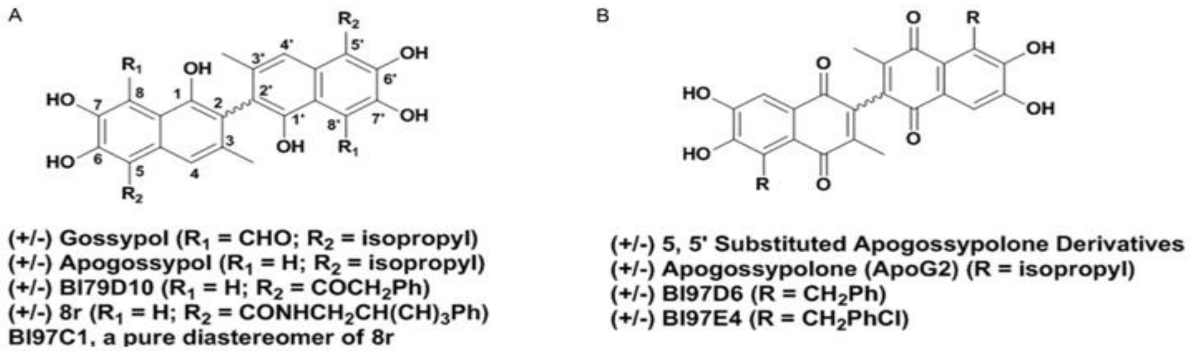

Supplementary Figure 3: Structure of (+/-) Gossypol and its derivatives; *A*. (+/-) Apogossypolone and its derivatives; *B*. (Published in *Frontiers in Oncology* 2011; **1**:28)

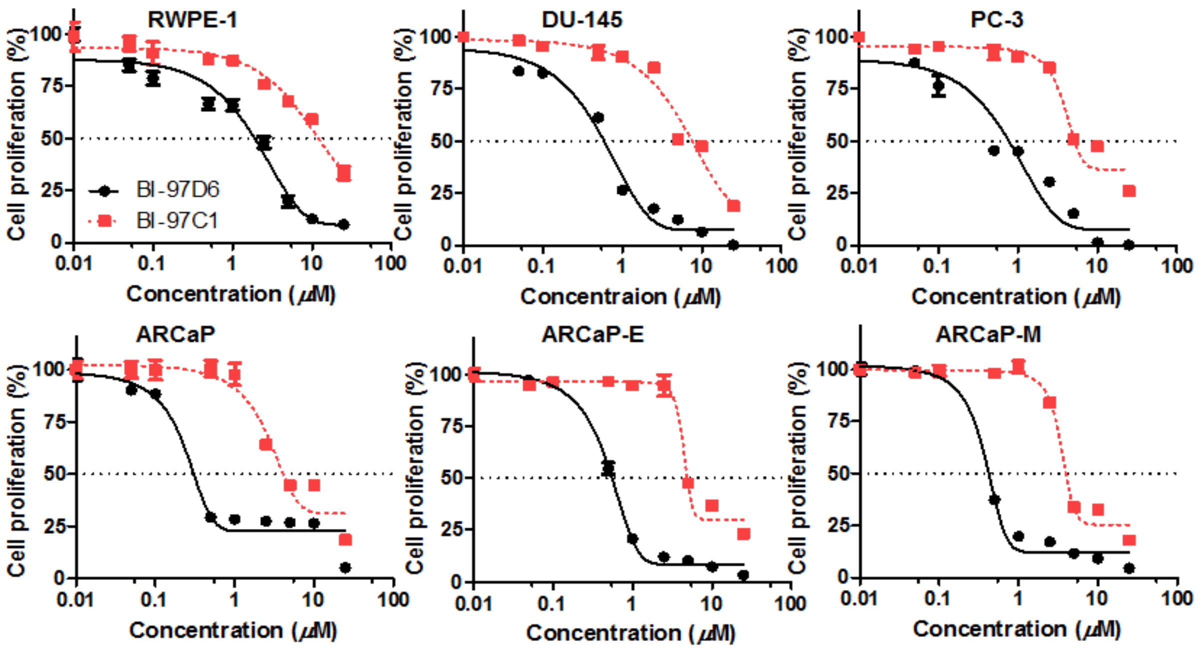

Supplementary Figure 4. Dose-response curve of Apogossypol derivative BI-97C1 and Apogossypolone derivative BI-97D6. CaP cells were treated with BI-97D6 and BI-97C1 for 72 h followed by MTT assays to determine growth.

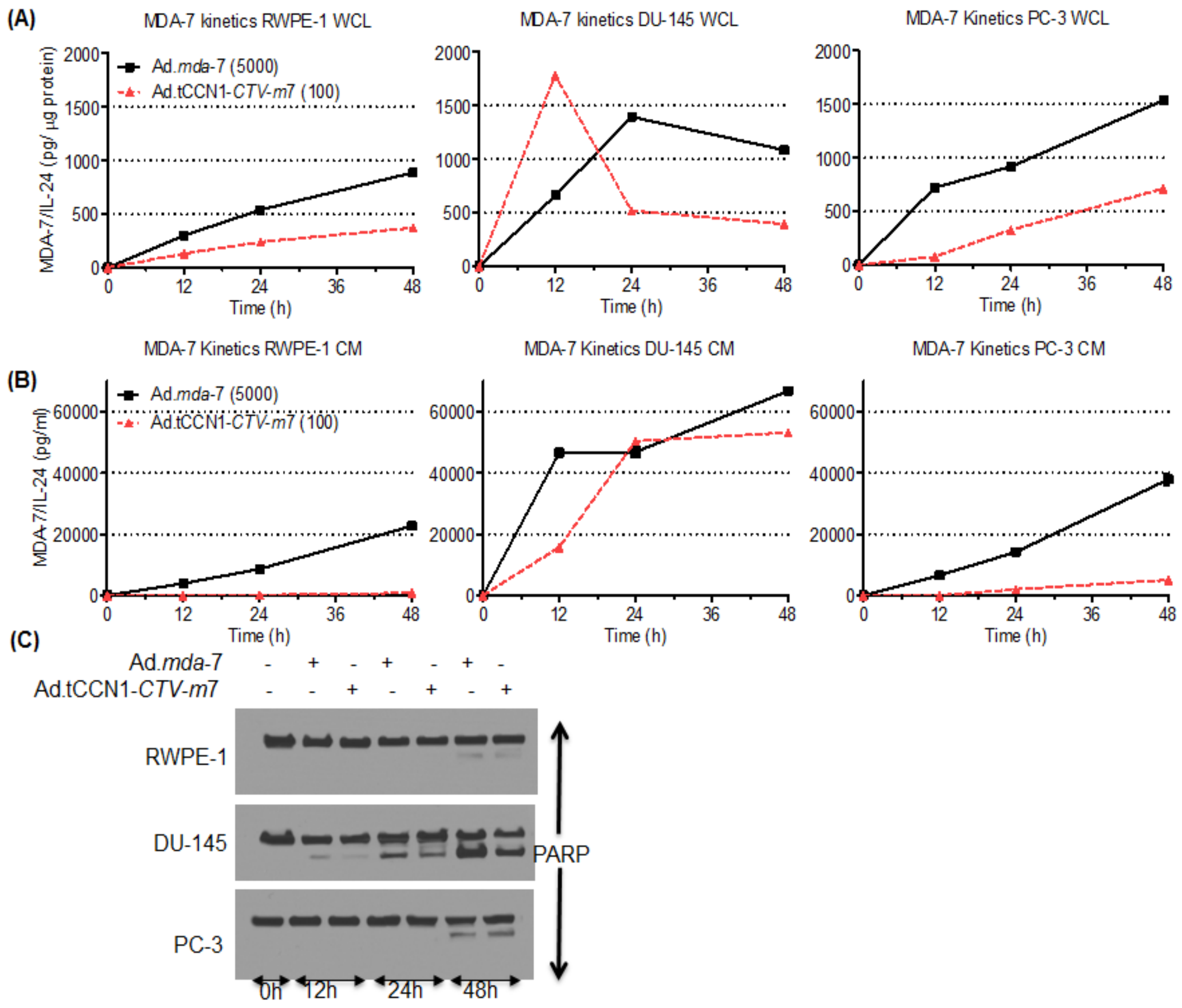

Supplementary Figure 5: Kinetics of MDA-7/IL-24 production in CaP cells after infection with Ad.*mda*-7 and Ad.tCCN1-*CTV-m*7. RWPE-1, DU-145 and PC-3 cells were infected with Ad.*mda*-7 and Ad.tCCN1-*CTV*-*m*7 at 5,000 and 100 vp/cell, respectively. Cells and conditioned medium (CM) was harvested at different time points post-infection with the Ads. Cells were lysed and centrifuged to collect whole cell lysates (WCL). Both ***A***; WCL and ***B***; CM were diluted and MDA-7/IL-24 levels were measured by using a h-IL-24 ELISA kit. ***C.*** Western blotting of WCL of RWPE-1, DU-145 and PC-3 cells infected with the indicated Ads with PARP cleavage serving as a temporal marker of apoptotic events.

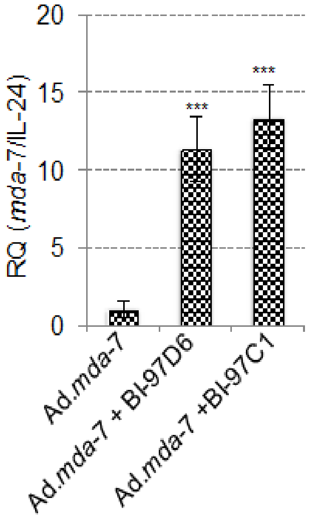

Supplementary Figure 6: BH3 mimetics BI-97D6 and BI-97C1 enhance the expression of *mda*-7/IL-24 mRNA. DU-145 cells were treated with 250 nM and 500 nM of BI-97D6 and BI-97C1, respectively, for 48 h and mRNA was isolated and cDNA was prepared followed by qPCR using a human *mda*-7/IL-24 and a GAPDH probe. p>0.001 (***) indicates significance level between Ad.*mda*-7 alone vs. Ad.*mda*-7 plus BI-97D6 or BI-97C1-treated cells.
